# Severe retinal hemorrhages at various levels with a serous retinal detachment in a pediatric patient with aplastic anemia–A case report

**DOI:** 10.3389/fmed.2023.1051089

**Published:** 2023-01-18

**Authors:** Xiaoshuang Jiang, Mengxi Shen, Licong Liang, Philip J. Rosenfeld, Fang Lu

**Affiliations:** ^1^Department of Ophthalmology, West China Hospital, Sichuan University, Chengdu, China; ^2^Department of Ophthalmology, Bascom Palmer Eye Institute, University of Miami Miller School of Medicine, Miami, FL, United States

**Keywords:** aplastic anemia, retinal hemorrhage, serous retinal detachment, vitrectomy, retinal atrophy, retinal edema

## Abstract

**Background:**

Aplastic anemia can cause ophthalmic abnormalities in patients. Vision loss in a child with aplastic anemia due to massive retinal hemorrhages at various levels is rare.

**Case presentation:**

A pediatric patient with aplastic anemia presented with retinal hemorrhages at multiple levels along with a serous retinal detachment in both eyes and subsequent retinal changes after pars plana vitrectomy.

**Conclusion:**

Anemia and thrombocytopenia in aplastic anemia could cause severe retinal hemorrhages and result in retinal atrophy and retinal edema. Vitrectomy can be performed to remove vitreous hemorrhage, but risk factors for retinal atrophy and edema need further investigation.

## Introduction

Aplastic anemia is a bone marrow failure syndrome with high mortality if untreated. Aplastic anemia commonly presents in patients between 15 and 25 years of age, with a second smaller peak after age 60. Patients with aplastic anemia may present with malaise and fatigue from severe anemia, hemorrhagic sequalae due to thrombocytopenia, and infection from neutropenia. Current treatment options for aplastic anemia include hematopoietic stem cell transplantation, immunosuppressive therapy, and supportive care (1).

Ophthalmic findings reported in aplastic anemia include conjunctival pallor, subconjunctival hemorrhage, orbital hematoma, hyphema, and retinal abnormalities. Specifically, cotton-wool spots, nerve fiber layer hemorrhages, central retinal vein occlusion, vitreous hemorrhage, retinal neovascularization, and serous retinal detachment have been described as abnormal retinal findings in aplastic anemia (2–9). Most of time, the retinal abnormalities need only observation without intervention. We are unaware of any reports about massive multi-level retinal hemorrhages with serous retinal detachment in pediatric aplastic anemia. We report here on the ophthalmic course of a pediatric patient with aplastic anemia who presented with severe retinal and vitreous hemorrhages with serous retinal detachment in both eyes and subsequent retinal changes after pars plana vitrectomy.

## Case presentation

**FIGURE 1:**
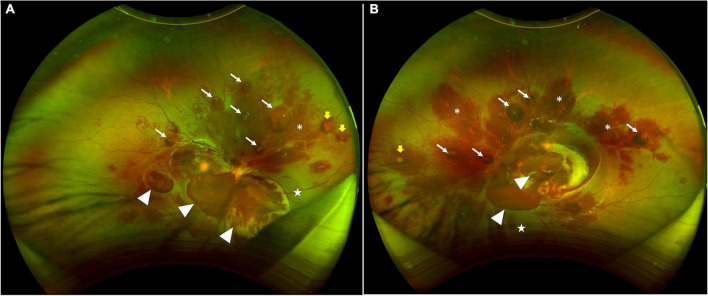
Scanning laser ophthalmoscopic images showing extensive dense preretinal, intraretinal, and subretinal hemorrhages in both retinas. **(A)** The right eye shows subhyloid hemorrhages (white arrows), hemorrhages between ILM and retina (white arrow heads), hemorrhages within the retina (asterisks), and sub-retinal hemorrhages (stars). Yellow arrows point at Roth spots. **(B)** The left eye shows subhyloid hemorrhages (white arrows), hemorrhages between ILM and retina (white arrow heads), hemorrhages within the retina (asterisks), at sub-retinal hemorrhages (stars). Yellow arrows point at Roth spots.

A 10-year-old boy with a history of aplastic anemia diagnosed in December 2019 presented to our clinic in December 2021 complaining of sudden vision loss in both eyes of 1 week duration without improvement. The patient denied any excessive physical exertion. Visual acuity was counting fingers at 20 cm in the right eye and hand movement in the left eye. Intraocular pressures were 10.2 mmHg in the right eye and 10.9 mmHg in the left eye using full auto tonometer (Canon, Japan, TX-20). The anterior segments of both eyes were quiet. Dilated funduscopic examination revealed extensive dense preretinal, intraretinal, and subretinal hemorrhages in both eyes (Figure 1). It was also noted that preretinal fibrosis was present along with hard exudates and Roth spots in retina.

Laboratory testing revealed hemoglobin of 81 g/L, leukocyte count of 1.79*10^9^/L, and platelets of 66*10^9^/L using the Coulter Prinicple (Systemx, Germany, XN-9000). Normal ranges are usually 115–150 g/L for hemoglobin, 3.5–9.5*10^9^/L for leukocyte count, 100–300*10^9^/L for platelets. Platelet counts varied between 72*10^9^/L and 81*10^9^/L before surgery after platelet transfusions.

**FIGURE 2:**
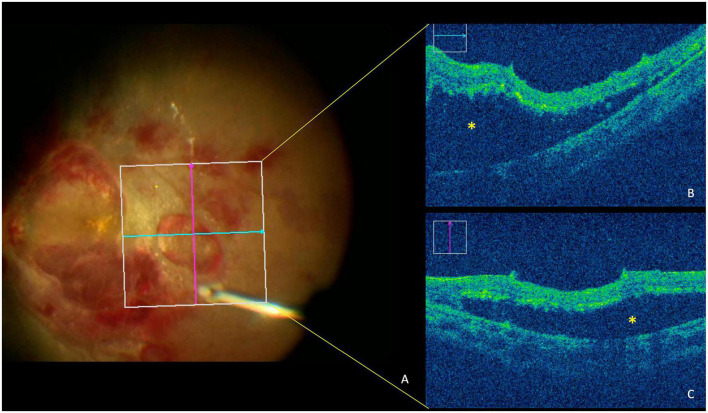
Optical coherence tomography (OCT) image during surgery shows serous retinal detachment in posterior fundus of the right eye. **(A)** Fundus imaging from the retina’s view during surgery. **(B)** OCT imaging shows the horizontal B-scan of retina. **(C)** OCT imaging shows vertical B-scan of retina. Asterisks denote detachment of the neuroretina from the retinal pigment epithelium.

Pars plana vitrectomy was performed to remove the vitreous haze and hemorrhage as well as the preretinal fibrosis 1 week after his presentation. Intraoperatively, several large and dense preretinal hemorrhages were removed. During the surgery, both intraretinal and subretinal exudation was observed along with a temporal neuroretinal detachment in the right eye as appreciated in the surgical optical coherence tomography (OCT) images (Rescan 700, Carl Zeiss Meditec, Dublin, CA, USA) (Figure 2). During surgery, the dense vitreous haze contained hemosiderin with blood clots adherent to the retina. This material was evacuated using the vitrectomy instrument. After the preretinal hemorrhages were removed, the temporal serous retinal detachment was observed in both eyes less than 1 month, and the laboratory testing revealed hemoglobin of 79 g/L, leukocyte count of 2.37*10^9^/L, and platelets of 72*10^9^/L.

**FIGURE 3:**
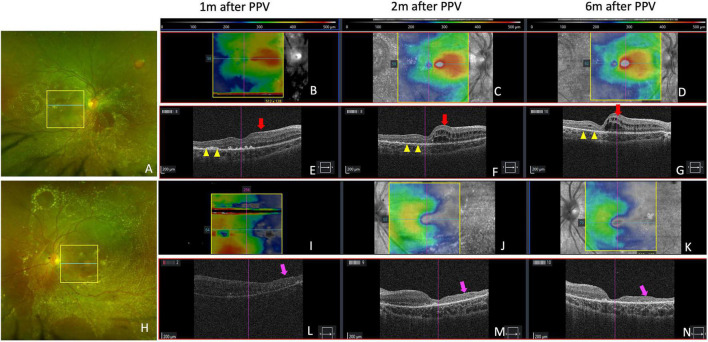
Retinal and optical coherence tomography (OCT) images showing retinal changes after vitrectomy. **(A,H)** Show retinal imaging by scanning laser ophthalmoscopy at 2 months. Yellow boxes in **(A,H)** show the area of OCT scanning in **(C,J)** with bule lines indicating the location of B-scans in **(F,M)**. **(B–D)** Show retinal thickness maps of right eye at 1 month, 2 months, and 6 months after vitrectomy. The color bars are shown above the panels. **(E–G)** Show horizontal OCT B-scans in the macular fovea of right eye. Yellow arrow heads show where hard exudates in the macular gradually absorbed without damage to outer retina. **(I–K)** Show retinal thickness maps of left eye at 1 month, 2 months, and 6 months after vitrectomy. **(L–N)** Show horizontal OCT B-scans in macular fovea of left eye. Red arrows show nasal macular edema after vitrectomy. Purple arrows show temporal macular atrophy.

At 6 months of follow-up, visual acuity was 10/20 in right eye and 20/200 in left eye. OCT imaging (Cirrus HD-OCT, Carl Zeiss Meditec, Dublin, CA, USA) was performed on both eyes at 1 month, 2 months, and 6 months after surgery. In right eye, hard exudates gradually absorbed in the macula without damage to the outer retina as seen on OCT imaging; however, macular edema appeared nasally 2 months after pars plana vitrectomy. In left eye, temporal macular atrophy was observed after reattachment of the retina (Figure 3).

## Discussion and conclusion

Anemia and thrombocytopenia were found to be important risk factors for developing hemorrhagic retinopathies. When anemia and thrombocytopenia were present at the same time, the frequency of retinopathy was 42% (10). In aplastic anemia, retinopathy was more frequently due to anemia and thrombocytopenia. This retinopathy was found in 69% of aplastic anemia patients who had Hb < 80 g/L and platelet counts of <50 × 10^9^/L. In a literature review of retinal findings in 200 patients with idiopathic aplastic anemia without any surgical treatment (bone marrow transplant), retinal hemorrhages were the most common reported manifestation which accounted for 56% of retinal abnormalities with subhyaloid/vitreous hemorrhages in 9%, peripheral retinal vasculopathy in 5.5%, and cotton-wool spots and optic disc edema in 4% each (9). Decreased visual acuity was usually due to preretinal and vitreous hemorrhages. In our case, vision impairment in both eyes was secondary to a large preretinal hemorrhage overlying the macular fovea.

The mechanism of retinal hemorrhages is likely to be multifactorial. Due to the presence of anemia in this disease, there is a compensatory increase in the cardiac output with increased turbulent flow that can lead to vascular endothelial damage and impairment of endothelial cell tight junctions, which may be responsible for the observed exudation and hemorrhage. Meanwhile, the impaired coagulation status in aplastic anemia due to a deficiency of platelets exacerbate the risk of hemorrhage. Observation without vitrectomy has been recommended for small vitreous and preretinal hemorrhages since most improve spontaneously, while vitrectomy should be performed to remove massive subhyloid and preretinal hemorrhages that impact vision since blood may cause permanent macular damage before it resolves.

Serous retinal detachment could also occur in pathological conditions that disrupt the integrity of blood-retinal barrier, which may be associated with inflammatory, infectious, infiltrative, neoplastic, vascular, and degenerative conditions (11). During the pars plana vitrectomy in our case, a serous retinal detachment was observed after the hemorrhage was removed. We speculated that ischemia in the retina and choroid impaired the blood-retinal barrier in our aplastic anemia patient, resulting in exudation from the retinal vasculature and the choroid. At the 6 month follow up visit, outer retinal atrophy and retinal edema were observed in the area of the exudative retinal detachment. We propose that the cystic macular edema results from the ischemia and hypoxia that still exists in the retina of this child patient.

In conclusion, this report illustrated a pediatric case of aplastic anemia presenting with sudden vision loss, significant retinal hemorrhages at various levels and a serous retinal detachment, which were successfully managed with vitrectomy. The identification of risk factors and the long-term prevention of retinal atrophy and edema require further investigation.

## Data availability statement

The raw data supporting the conclusions of this article will be made available by the authors, without undue reservation.

## Author contributions

XJ performed the literature search, collection, and drafted the manuscript. MS contributed to image and photo illustration. LL contributed to the data collection. PR edited the manuscript and gave consultation. FL completed the all examination, confirmed the diagnosis, and performed the vitrectomy. All authors read, edited, and approved the final version of the manuscript.
